# Closing the treatment gap for alcohol use disorders in low- and middle-income countries

**DOI:** 10.1017/gmh.2022.57

**Published:** 2022-12-09

**Authors:** Abhijit Nadkarni, Yashi Gandhi, Urvita Bhatia, Richard Velleman

**Affiliations:** 1 Centre for Global Mental Health, Department of Population Health, London School of Hygiene and Tropical Medicine, London, UK; 2 Addictions Research Group, Sangath, Porvorim, India; 3 Department of Psychology, University of Bath, Bath, UK

**Keywords:** alcohol, low-income countries, developing countries, interventions

## Abstract

The alcohol-attributable disease burden is greater in low- and middle-income countries (LMICs) as compared to high-income countries. Despite the effectiveness of interventions such as health promotion and education, brief interventions, psychological treatments, family-focused interventions, and biomedical treatments, access to evidence-based care for alcohol use disorders (AUDs) in LMICs is limited. This can be explained by poor access to general health and mental health care, limited availability of relevant clinical skills among health care providers, lack of political will and/or financial resources, historical stigma and discrimination against people with AUDs, and poor planning and implementation of policies. Access to care for AUDs in LMICs could be improved through evidence-based strategies such as designing innovative, local and culturally acceptable solutions, health system strengthening by adopting a collaborative stepped care approach, horizontal integration of care into existing models of care (e.g., HIV care), task sharing to optimise limited human resources, working with families of individuals with AUD, and leveraging technology-enabled interventions. Moving ahead, research, policy and practice in LMICs need to focus on evidence-based decision-making, responsiveness to context and culture, working collaboratively with a range of stakeholders to design and implement interventions, identifying upstream social determinants of AUDs, developing and evaluating policy interventions such as increased taxation on alcohol, and developing services for special populations (e.g., adolescents) with AUDs.

## Impact statement

Despite the huge burden of alcohol use and alcohol use disorders (AUDs) in terms of morbidity and mortality, access to appropriate and evidence-based care is limited. The treatment gap is particularly magnified in low- and middle-income countries (LMICs). Our review examines the size and causes of this large treatment gap in LMICs. More importantly, we identify innovative models of care that could be implemented in LMICs to reduce the treatment gap for AUDs. Finally, we end with a reflection on future perspectives which could shape research, policy and practice aimed at increasing access to evidence-based and contextually relevant care for AUDs in LMICs and other low-resource settings.

## Social media summary

Critical ingredients 4 increasing access 2 care for those with drinking problems in developing world-evidence-based decision-making, cultural/contextual responsiveness of interventions designed & implemented collaboratively with key stakeholders & focuson policy interventions.

## Introduction

The morbidity and mortality attributed to alcohol use is high across the world but vary considerably between regions and countries. The highest alcohol-attributable burden, estimated as disability-adjusted life years (DALYs) per 100,000 people, is in Eastern Europe and Southern sub-Saharan Africa (Degenhardt et al., 2018). The countries with the highest alcohol-attributable DALYs per 100,000 people include Russia, Ukraine, and Belarus in East Europe, and Lesotho, Central African Republic, and Burundi in sub-Saharan Africa (Degenhardt et al., 2018). However, in terms of absolute numbers, the highest alcohol-attributable DALYs are in East Asia, South Asia, Eastern Europe, and Tropical Latin America (Degenhardt et al., 2018).

Alcohol use disorders (AUDs) are commonly operationalised using the Diagnostic and Statistical Manual of Mental Disorders (DSM) and the International Classification of Diseases (ICD). They are characterised by impaired control over alcohol consumption resulting in continued, heavy, compulsive, and often escalating, alcohol use despite detrimental psychological, biological, behavioural, or social consequences for themselves, their family members and friends, and society in general (Carvalho et al., 2019).

AUDs are widely prevalent, affecting 5.1% (95% CI 4.9–5.4) globally – 8.6% (95% CI 8.1–9.1) men and 1.7% (95% CI 1.6–1.9) women (World Health, 2018; Rehm and Shield, 2019). The prevalence of AUDs is highest in high-income countries (8.4%, 95% CI 8.0–8.9) and upper-middle-income countries (5.4%, 5.0–6.0) (World Health, 2018).

AUDs are associated with high morbidity and mortality; with the excess mortality associated with AUDs resulting in life expectancy that is lower by more than 20 years from the population average (Samokhvalov et al., 2010; Westman et al., 2015; Schwarzinger et al., 2018). Global DALYs attributable to alcohol use are highest for injuries (21.0 million DALYs), cardiovascular diseases (20.8 million DALYs), and cancers (14.8 million DALYs) (Degenhardt et al., 2018). Overall, 2.8 million deaths across the world in 2016 were attributed to alcohol use and AUDs (Degenhardt et al., 2018).

The leading causes of alcohol-associated deaths are cardiovascular disease, diabetes, injuries, gastrointestinal diseases, and cancers (World Health Organization, 2014). However, harms from alcohol use are not restricted only to health but comprise other domains of life such as violence, crime and loss of productivity (Martin, 2001; Thavorncharoensap et al., 2009). The global economic costs of alcohol consumption are estimated to be 1,306$ per adult or 2.6% of the GDP; and while 39% of these are incurred through direct costs (e.g., healthcare), the majority of costs are through indirect costs such as loss of productivity (61%; Manthey et al., 2021).

Even in high-income countries, alcohol use and AUDs lead to greater harm per litre of alcohol consumed in those from low socioeconomic strata compared to those from the high socioeconomic strata (Collins, 2016). More specifically, individuals with a low socioeconomic status are at least twice as likely to die from heavy alcohol use than those with high socioeconomic status (Probst et al., 2015). This is particularly important for low- and middle-income countries (LMICs) where a significantly large proportion of the population resides in impoverished conditions.

There is good evidence of a range of psychosocial and pharmacological interventions for effective treatment of AUDs in a variety of settings (Carvalho et al., 2019). In addition, evidence from high-income countries indicates that alcohol control policies of restricting availability, banning marketing, and increasing taxation are highly cost-effective in reducing alcohol-attributable harm (Carvalho et al., 2019). It is estimated that increasing access to treatment within primary care settings for 30% of heavy-drinking patients could decrease the overall prevalence of harmful use of alcohol by 10–15% and reduce the incidence of AUD by 5–14% (Sassi, 2015). Similarly, if the proportion of patients with AUD who received treatment doubled, the annual incidence of AUD would decrease to between 1 and 4% (Sassi, 2015). Finally, if 60% of people with AUD were treated with effective interventions, approximately 13% of male and 9% of female alcohol-attributable deaths would be averted in a 12-month period (Rehm et al., 2013).

With alcohol consumption stabilising or reducing in developed countries, transnational corporations have identified LMICs for sales growth, and hence it is expected that both overall consumption and harmful drinking will rise in such countries. For example, the African continent is seen as a major opportunity for market expansion as almost 70% of the adult population is abstinent, and hence there are expected increases in the number of potential new alcohol consumers, especially young people and women (World Health Organization, 2014; Toesland, 2016). Hence, the aim of this paper is to focus on LMICs to examine the burden of AUDs, the magnitude of the treatment gap, key contributors to the treatment gap, the existing health systems response to the treatment gap, and innovative solutions to reduce the treatment gap. In the following sections, each one of these objectives is discussed in greater detail.

## Burden of AUDs in LMICs

There is a moderately negative relation between gross national income (GNI) and amount of harm caused due to alcohol (World Health, 2018). In other words, the alcohol-attributable disease burden is found to be greater in LMICs as compared to high-income countries. The age-standardised alcohol-attributable burden of disease and injury is highest in Africa (70.6 deaths and 3,044 DALYs per 100,000) although alcohol consumption is highest in the European region (World Health, 2018). The consumption patterns are different as well – alcohol is consumed mostly by younger men (83%) in LMICs like Belarus, Brazil, Mexico and India, compared to middle-aged men and women in HICs (Smyth et al., 2015; World Health, 2018).

There are geographical variations within LMICs as well. The BRICS (Brazil–Russia–India–China–South Africa) countries are some of the world’s largest countries, both with regard to population size and land coverage. From 1990 to 2013, there was an overall decrease of alcohol-attributable age-standardised DALYs in Brazil, China and South Africa, and an overall increase in Russia and India (Rabiee et al., 2017).

There is also evidence on the role of socioeconomic factors on harmful drinking patterns even within LMICs. In South Africa, high-income earners had the highest drinking prevalence but low-income earners consumed more alcohol on average, spent a greater proportion of household income on alcohol and experienced a higher burden of alcohol-related harm (Walls et al., 2020). Furthermore, in South Africa, 60% of all alcohol-attributable deaths occurred in the lower 30% of the socioeconomic distribution (World Health, 2018).

Overall, the premature mortality rate is disproportionately higher in LMICs with more than 85% of all deaths attributable to alcohol occurring in these countries. This is partly because of riskier patterns of consumption, and the lack of safe ‘built’ environments like roads which increases the risk of injuries in these countries (Patel et al., 2016). The alcohol-attributable burden of disease is also greater in LMICs due to the larger burden caused by associated conditions such as tuberculosis, cardiovascular diseases, interpersonal violence, self-harm and poisoning, and unintentional injuries (Patel et al., 2016; World Health, 2018). While this is true worldwide, its burden and impact are more pronounced in LMICs primarily since these behaviours are linked to AUDs which are rarely identified within the primary health care system. Moreover, while the rates of alcohol consumption among women in LMICs are lower, they suffer from disproportionately greater social consequences per litre consumed due to cultural norms (Patel et al., 2016). Overall, the health and societal costs accrued due to alcohol consumption outweigh any economic benefits resulting from the alcohol industry in LMICs (Patel et al., 2016).

## Interventions for AUDs

The evidence about treatment interventions for AUDs in LMICs is limited and summarised in this section. In this section, we will briefly summarise universal interventions such as health promotion, brief interventions (BIs), psychological treatments, family interventions, and biomedical treatments. Some of these interventions have been described in much more detail in other reviews which can supplement some of the evidence presented in our review (Joseph and Basu, 2017; Sileo et al., 2021; Ghosh et al., 2022; Staton et al., 2022).

### Health promotion and education

Although several RCTs have evaluated health promotion and education interventions in a range of settings such as the workplace, the community, schools, and clinics (Kalichman et al., 2008; Chhabra et al., 2010; Cubbins et al., 2012; Aira et al., 2013; Bolton et al., 2014; Marsiglia et al., 2015; Rotheram-Borus et al., 2015; Chaudhury et al., 2016; Jordans et al., 2019), a majority of the programs addressed alcohol use in the context of HIV/AIDS prevention and risk reduction (Kalichman et al., 2008; Chhabra et al., 2010; Cubbins et al., 2012). Some of these studies reported positive results (Kalichman et al., 2008; Aira et al., 2013; Marsiglia et al., 2015; Chaudhury et al., 2016), while others had mixed results, that is, change in some outcomes and not in others or an initial reduction in alcohol use followed by a relapse (Aira et al., 2013; Rotheram-Borus et al., 2015).

### Brief interventions

BIs are generally characterised by a few short sessions involving an assessment of individual risk with feedback and advice, followed by provision of structured advice, or brief motivational interviewing that takes a more patient-centred approach, or a combination of both (Heather, 2010). BIs are the most tested interventions for AUDs in LMICs, most commonly using motivational interviewing techniques (Pengpid et al., 2013; Signor et al., 2013; Ward et al., 2015; Kamal et al., 2020); delivered by non-specialist health workers (NSHWs; Noknoy et al., 2010; Mertens et al., 2014) or through digital platforms (Baldin et al., 2018; Bedendo et al., 2019). There is substantial evidence on the effectiveness of BIs on a range of short- and long-term drinking outcomes, in healthcare and community settings, in men as well as women, and when delivered by NSHWs, or digitally (Noknoy et al., 2010; Pengpid et al., 2013; de Oliveira Christoff and Boerngen-Lacerda, 2015; Bedendo et al., 2019, 2020; Wechsberg et al., 2019). Compared to a range of other public health policies designed to reduce alcohol-related harm (e.g., regulation of alcohol advertising) BIs achieve larger effects as measured by DALYs (Franco, 2015).

### Psychological treatments

Compared to BIs, the evidence about psychological treatments (brief or extended) for AUDs in LMICs is limited. Most of the existing evidence is about interventions based on motivational interviewing techniques or cognitive behavioural therapy (CBT) delivered to participants in a range of settings such as hospitals, emergency departments, primary care, and specialist clinics for patients with HIV or tuberculosis (Nattala et al., 2010; Shin et al., 2013; Daengthoen et al., 2014; Nadkarni et al., 2017b; Papas et al., 2021). Most randomised controlled trials (RCTs) demonstrate the effectiveness of interventions such as combination therapy, dyadic intervention, motivational interviewing with or without problem-solving, and CBT (Nattala et al., 2010; Rendall‐Mkosi et al., 2013; Daengthoen et al., 2014; Sorsdahl et al., 2015; Madhombiro et al., 2020; Papas et al., 2021).

BIs and psychological treatments could be conceptualised as lying on a continuum of care. They can be distinguished from each other based on several criteria, including the outcomes they try to achieve. For example, BIs are generally focused on motivating the drinker to initiate change (e.g., enter treatment) while psychological treatments address larger concerns (e.g., addressing long-standing problems that exacerbate alcohol use) (Center for Substance Abuse Treatment, 1999). Other characteristics that might distinguish them include the number and duration of the sessions (fewer and shorter sessions for BIs), delivery settings (non-traditional treatment settings such as a social service or primary care), and delivery agents (BIs delivered by non-specialists; Center for Substance Use Treatment, 1999).

### Family-focused interventions

Traditionally, family members have been neglected in addictions services, with the focus of treatments largely being limited to engaging the person with drinking problems (Orford et al., 2013). Over the years, a number of psychotherapeutic approaches have been designed and evaluated where family members are engaged jointly with the person with drinking problems, or in their own right. A recent meta-analysis highlighted the effectiveness of psychosocial interventions directed at affected family members in improving clinical, health and relationship outcomes in family members and treatment engagement in the person with AUD (Merkouris et al., 2022).

### Biomedical treatments

Some RCTs evaluated biomedical treatments such as medications (e.g., naltrexone, gabapentin, disulfiram, and topiramate), combined behavioural and medication interventions (e.g., acamprosate with Alcoholics Anonymous, baclofen with a BI), and brain stimulation. Naltrexone had a limited impact on drinking outcomes (Ahmadi et al., 2004; Shin et al., 2013), topiramate had mixed results (Baltieri et al., 2008; Likhitsathian et al., 2013), and gabapentin, acamprosate, and baclofen showed positive results (Furieri and Nakamura-Palacios, 2007; Baltieri et al., 2008; Gupta et al., 2017). The few studies that tested the effectiveness of transcranial direct current stimulation (all in Brazil) showed mixed results with some studies reporting positive reports (Boggio et al., 2008; da Silva et al., 2013), and others with positive effects on some outcomes and not on others (Klauss et al., 2014).

## Treatment gap in LMICs

Treatment gap refers to the proportion of individuals who require treatment for a particular condition but do not receive it; and this is an important metric of the inequitable supply of services and the presence of disparities in both the needs and demands for treatment (Patel et al., 2010). Globally the treatment gap for people with mental disorders represents a major public health challenge as demonstrated by data from community surveys in 25 countries in the WHO World Mental Health Survey Initiative (Kessler et al., 2009). Only 14% of individuals with mental disorders in lower-middle-income countries, 22% in upper-middle-income countries, and 37% in high-income countries received treatment (Evans-Lacko et al., 2018). The highest treatment rate (18%) was in the general medical sector followed by the specialist mental health sector (14%); while the treatment rates were much lower in the human services sector (e.g., religious or spiritual advisor, social worker) and complementary alternative medicine sector (4% each) (Evans-Lacko et al., 2018).

Despite the high burden of AUDs and availability of evidence-based interventions, outlined above, access to appropriate treatment remains low. The pooled treatment rate of AUD from any source of treatment is 17.3% (95% CI 12.8–22.3), that is, a treatment gap of 82.7% (Mekonen et al., 2021). This effectively means that four out of five individuals with AUD do not have access to appropriate care for their drinking problems. The treatment rate varies widely between countries – 3.5% in Uganda to 51.8% in the United Kingdom; and overall the treatment rate of 9.3% (95% CI: 4.0–15.7%) in LMICs is much lower than the overall global figure (Mekonen et al., 2021).

Similarly, despite the evidence about the effectiveness of a range of interventions for AUDs in LMICs, the ‘treatment gap’ remains substantial. Mental Health Care Gap is proposed as a more comprehensive measure to describe access to care as this encompasses ‘treatment gap’, ‘psychosocial care gap’ (lack of psychosocial interventions), and ‘physical health care gap’ (lack of or substandard provision of physical health interventions) (Pathare et al., 2018). However, in the absence of reliable estimates of this new metric, we will focus on the conventional treatment gap to illustrate poor access to care for AUDs in LMICs.

Research on the treatment gap for AUDs in LMICs is limited, but sufficient to conclude that overall a very small proportion of people with AUDs have access to relevant care and this varies between countries. In a study that conducted health-facility-based cross-sectional studies in five LMIC districts, among participants who screened positive for AUD, clinical detection of AUD ranged from 0% in Ethiopia and India to 7.8% in Nepal (Rathod et al., 2018). Additionally, treatment access was 0% in all those countries except Nepal, where it was 2.2% (Rathod et al., 2018). In the same study, contact with any kind of relevant (but not necessarily evidence-based) treatment over the past 12 months (‘contact coverage’), for adults with probable AUDs, ranged from 2.8% in India to 5.1% in Nepal (Rathod et al., 2016); and in Ethiopia, lifetime contact coverage for probable AUD was 13.1% (Rathod et al., 2016). Finally, relatively older data indicates that the treatment gap for AUDs was high in LMICs such as Brazil (53.3%), Mexico (93.8%), and Turkey (89.8%) (Kohn et al., 2004). Although this data is relatively old, there is nothing to indicate that these treatment gaps would have decreased substantially over the years.

## Contributors to the treatment gap in LMICs

The treatment gap may be explained by some combination of (1) limited access to general health and mental health care, (2) poor accessibility of evidence-based treatments, (3) limited availability of and clinical skills among health care providers, (4) lack of political will and/or financial resources, (5) historical stigma and discrimination against people with AUDs, and (6) poor planning and implementation of policies (Connery et al., 2020).

Stigma towards people with AUDs contributes to marginalisation of such individuals, self-stigma, avoidance of help-seeking, social isolation, and lack of awareness among policymakers and clinicians about the availability of effective treatments (Connery et al., 2020). The relationship between stigma towards people with AUDs and help-seeking has not been extensively studied in LMICs. The available evidence shows that there is a high internalised stigma experienced by those with AUD and the fear of being labelled an ‘alcoholic’ leads to low utilisation of treatment services, since it confirms their membership of the stigmatised group (Zewdu et al., 2019). Research from India and Uganda indicates that, people with AUDs feel disappointed with themselves, feel embarrassed and ashamed, believe that others think that they cannot achieve much in life because of their alcohol problems, are ignored by people or taken less seriously because of their problems, and feel out of place in the world (Rathod et al., 2015; Nalwadda et al., 2018); all of which could result in a reluctance to access help. Other common factors that might hinder service utilisation include low perceived needs, lack of awareness about the available services, inability to afford the treatment cost, and limited access to effective treatments (Edlund et al., 2006; Saraceno et al., 2007; Luitel et al., 2017).

In most LMICs, interventions for AUDs are expected to be delivered by mental health care workers. However, estimates in 2005 indicate that there was a shortage of 1.2 million mental health workers (Kakuma et al., 2011) in LMICs, and there have not been any drastic systemic changes over the years to indicate that this shortfall might have reduced significantly. In such circumstances, primary care practitioners can potentially be frontline providers of care for those with AUDs. However, challenges in primary care that pose barriers to delivering AUD care in LMICs include limited training, high clinical workload, competing clinical priorities, and perceived complexity of interventions for AUDs (Myers et al., 2012; Rathod et al., 2017; Ronzani et al., 2019).

Finally, explanatory models of ill health are closely linked to the cultural context (Jacob and Patel, 2014). Additionally, access to appropriate care is mediated by cultural practices and traditions, which might sometimes prevent people from accessing treatment (Bracke et al., 2019). Interventions which are not relevant to the context and do not incorporate the nature of the social, economic and cultural environment might not be acceptable and hence not effective in reducing the treatment gap.

## Health system response

The costs to plug the gaps in the health systems to increase access to care for AUD are not substantial. For example, a package that will achieve coverage levels of 80% of cases with psychosis and bipolar disorder, and a modest 25–33% of cases with depression and risky drinking would cost only 2$ per capita in low-income countries, and 3–4$ in middle-income countries (Levin and Chisholm, 2016). Another estimate calculated that the annual cost of delivering a package of interventions for schizophrenia, depression, epilepsy, and AUDs in Sub-Saharan Africa and South Asia would be 3–4$ per capita (Levin and Chisholm, 2016).

However, the size and character of models of care for AUD that a country adopts depend on how it views the alcohol problems in its population and its reliance on alcohol-related revenue, rather than on cost-effectiveness of the model, the treatment requirements of the country, and the availability of economic resources (Babor et al., 2008). In many LMICs, the service models adopted for AUDs are mainly organised around tertiary care interventions, focus only on treatment of alcohol dependence, and have a disproportionate emphasis on long-term residential rehabilitation, specialised clinics, and psychiatric hospitals (Perngparn et al., 2008). Even these services are poorly accessible as they are inequitably distributed, primarily situated in urban areas, and often run by private providers charging fees which are unlikely to be affordable to all. Most people with early alcohol-related problems consult primary health care clinicians, mostly for physical health problems related to alcohol use. However, in the absence of routine screening for AUD in primary care and lack of training to recognise the problem, most people with AUDs in LMICs remain untreated for over a decade (Benegal et al., 2009). In summary, in many LMICs, prevention in earlier stages of problem drinking is mostly non-existent and alcohol-related problems are first addressed when they are already severe and difficult to treat.

## Innovations to improve access to care for AUDs in LMICs

### Designing local and culturally acceptable solutions

Besides individual risk factors for AUD (e.g., male gender, lower education level, unemployment), there are critical contextual forces such as availability of alcohol, alcohol advertising, policies related to alcohol availability, and norms around drinking, that shape drinking behaviours (Gruenewald et al., 1993; Alaniz, 1998; Borsari and Carey, 2001; Gruenewald et al., 2002). Hence, bridging the treatment gap will require making strategic choices about evidence-based treatments after suitable adaptation to suit the context, and special attention being paid to local patterns of drinking and its intersection with cultural influences (e.g., boatmen of Benares in India, while averse to any public displays of drunkenness on the sacred ghats recognise alcohol as a legitimate source of relaxation [Doron, 2010]), and existing health systems. This requires a detailed landscaping of the context and this could be achieved through a situational analysis involving key stakeholders who will be able to provide critical information about which interventions will be acceptable, and also on implications for delivery. Such a participatory exercise affords additional advantages such as reduction of stigma and promoting buy-in through dialogue about concerns and potential solutions to address them. It also allows to pre-empt potential barriers which could consign a new initiative to failure. For example, pharmacologic interventions may not be best suited for settings with weak supply chains, and the choice of psychological treatments will depend on acceptability of ‘talking treatments’ to the target group, as well as systemic considerations such as the number and skills of health care providers. Although existing evidence is limited, culturally adapted interventions for AUDs are a promising approach for reducing alcohol use and related consequences with a demonstrated effect size of 0.25 (95% CI 0.08, 0.43) (Hai et al., 2021). Case study 1 is an example of the importance of contextual adaptations to make program relevant to the settings in which it is being delivered.

Case study 1: The Healthy Women Healthy Living (HWHL) is an intervention developed for reducing heavy drinking in women living with HIV in the US. The HWHL was adapted for use in Uganda (Leddy et al., 2021) through identification of core intervention elements to be retained, and ‘surface structure’ adaptations of the content, focus group discussions with key stakeholders including patients, and cognitive interviews with patients and patient helpers. This process resulted in adaptations to account for varying literacy levels in the setting, acknowledgement of motivations for reducing alcohol consumption that include salient concepts such as retaining the respect of family, shifting of focus from alcohol use in one’s home to reflect the social nature of drinking and peer pressure to drink in Uganda, and inclusion of culturally relevant behavioural strategies to reduce alcohol use, such as drinking tea instead of alcohol.

Several models have been proposed to guide contextual adaptations to complex interventions and they appear to have several convergence points (Barrera and Castro, 2006; McKleroy et al., 2006; Kumpfer et al., 2008; Wingood and DiClemente, 2008; Nadkarni et al., 2015). The defining feature of these models is that they integrate existing theory and procedures (‘top-down’ elements), with input from contextually relevant stakeholder groups (‘bottom-up’ elements) to arrive at an adapted version that can then be rigorously evaluated.

### Health system strengthening

Many people with AUDs do not come into contact with addiction services, until the condition is severe. Hence, interventions need to be made available through alternative delivery platforms to reach those individuals who are not accessing routine healthcare systems. Such an integration requires buy-in from a range of relevant stakeholders and would involve sensitisation of leaders and front-line providers from such programs to the relevance of treating AUDs and of embracing accountability for individuals with AUDs who are traditionally excluded from health care. Thus, reducing the treatment gap for AUDs requires multidisciplinary effort with specialists such as psychiatrists and psychologists, working collaboratively with primary care providers, healthcare teams focused on medical conditions commonly co-occurring with AUDs, community-based health and social workers, and peer and lay counsellors.

The general principles guiding such health system strengthening include designing the program through participatory planning with multiple stakeholders, using contextually relevant screening tools for early identification of AUDs in routine care, training health and other workers in the use of manualised and brief low-intensity psychological interventions, providing supervision and support, and rapidly responding to natural opportunities in which political will or funding can be leveraged to strengthen AUD care (Davies and Lund, 2017).

Collaborative stepped care is one such health system-strengthening approach which enhances health system efficiency in patients with complex and chronic problems such as AUDs (Kodner and Spreeuwenberg, 2002). In such an approach, patients start treatment with low-intensity, low-cost interventions, and, guided by systematically monitored treatment outcomes, move to a higher-intensity treatment only if necessary. This allows for maximising efficiency by deploying limited resources according to need, and reserving highly specialised, intensive, and expensive resources for those with the most complex or severe problems.

The collaborative stepped-care approach has been most successfully used in high-income countries for treating common mental disorders in primary care, with some evidence from LMICs such as India (Archer et al., 2012). In addition to such evidence from highly controlled research studies, there are case studies from LMICs such as Brazil and India which demonstrate successful real-world implementation of the collaborative stepped-care approach for provision of mental healthcare (Shidhaye et al., 2015). Case study 2 describes a program designed to increase access to care for AUD through task-sharing with lay-counsellors based in primary care settings.

Case study 2: Counselling for alcohol problems (CAP) is an example of a contextually adapted brief psychological treatment for harmful drinking that is designed to be delivered through task-sharing with non-specialist health workers in primary care settings. CAP is a 3-phase treatment delivered over 1 to 4 sessions based on a motivational interviewing stance and involves the following strategies: assessment and personalised feedback, family engagement, drink refusal skills, skills to address drinking urges, problem-solving skills and handling difficult emotions, and relapse prevention and management (Nadkarni et al., 2015). CAP was tested in a trial in India and was found to be superior to enhanced usual care in reducing drinking (Nadkarni et al., 2017a,b). It was also found to be cost-effective, which makes it a potentially key strategy to reduce the treatment gap for AUD.

There is no reason why these successful models could not be extended to AUDs by systematic identification in primary care, close involvement of patients in joint decision-making regarding their care, development of a holistic care plan that includes psychological interventions, social care, and medication management where appropriate, streamlined referral pathways, regular and planned monitoring of patients, and consultation with specialists for patients who do not show clinical improvement.

### Horizontal integration of care into existing models of care

In many global health settings, there are existing robust healthcare delivery platforms that are potentially well-suited to integrate AUD identification and treatment. These include programs such as those for HIV and TB which are a natural fit for layering on treatments for AUD because of the strong causal relationships between these conditions. Additionally, such communicable diseases programs are typically high on the priority agenda even in LMICs and hence there is already existing strong buy-in from policymakers. Integrating AUD interventions into these existing healthcare platforms helps leverage available health systems such as medication supply chains and health worker cadres (Shidhaye et al., 2015). Case study 3 describes the efficient use of limited resources through the integration of AUD care in an existing program which provides services for a clinical condition which is strongly associated with AUDs.

Case study 3: A culturally adapted cognitive-behavioural therapy (CBT) intervention was integrated into an HIV outpatient clinic in Kenya. It was delivered by paraprofessional counsellors to HIV-positive patients with heavy drinking. The intervention was delivered over six sessions and included analysis of behaviour, skills for coping with triggers, urges and high–risk situations, identifying risky decisions leading to drinking, problem-solving skills, drink refusal skills, and relapse prevention strategies (Papas et al., 2010). The CBT intervention was found to be superior to healthy lifestyle education in reducing alcohol use (Papas et al., 2021).

The success of the collaborative stepped care model and horizontal integration of care into existing health care systems, described above, hinges on a ‘case manager’ who is responsible for coordinating care and who forms the critical link between patients, their families, primary care, and specialist health services (Patel et al., 2010). Such a resource does not routinely exist in low-resource settings and funding needs to be leveraged for identifying appropriate individuals and building their capacities to play such a role.

### Task sharing to optimise limited human resources

Task-sharing is a human resource innovation which involves strategic redistribution of some specialist tasks to appropriately trained and supervised non-specialist workers to increase access to evidence-based care through efficient use of limited resources (Shifting, 2008). Given the shortage of specialist healthcare workers and the high workload on existing primary care services in LMICs, access to care for AUD necessitates task-sharing with non-medical lay staff, community stakeholders as well as family members. Evidence from LMICs demonstrates that task-sharing is effective in supporting recovery from depression and anxiety, reducing symptoms of perinatal depression, reducing symptoms of adults with post-traumatic stress disorder, improving day-to-day functioning of people with schizophrenia, improving the behavioural symptoms of people with dementia and the mental well-being, burden and distress of carers of people with dementia (van Ginneken et al., 2021).

Trained non-specialist workers can play a crucial role in identifying, engaging with, and building awareness in individuals with AUDs and their family members, maintaining follow-up, ensuring adherence, monitoring clinical outcomes, and delivering low-intensity, evidence-based, first-line psychological treatments such as motivational interviewing (van Ginneken et al., 2021). Building successful task-sharing models requires the identification of shared needs and goals, mutual and respectful engagement, robust training and ongoing supervision in evidence-based treatment protocols, and quality control through monitoring and iterative feedback loops to optimise services. Although this requires intensive front-loading of resources, in the longer term it results in significant gains in increasing access to sustainable evidence-based care. Evidence on task-shared interventions for AUDs in LMICs is growing and indicates their effectiveness in reducing risk of, and increasing recovery from, hazardous and harmful alcohol use (van Ginneken et al., 2021). The characteristics of BIs (e.g., short, opportunistically target drinkers who are not seeking help for their drinking problems) make them a particularly appealing choice for task-sharing in low-resource settings.

### Working with families

Families are adversely affected by AUDs, and they also play a critical role in the development of, as well as recovery from, AUDs (Copello et al., 2005). Additionally, in LMICs, traditional family structures, prevailing stigma around AUDs, and cultural beliefs held by family members can influence help-seeking, and also negatively influence recovery processes (Kumar et al., 2022).

Hence, the involvement of family members in the treatment of AUDs, especially in socio-centric LMICs, is critical. The engagement with family members can be through three pathways: (1) working with family members to promote the entry and engagement of the person with AUD into treatment; (2) joint involvement of family members and drinker in the treatment of the latter and (3) interventions responding to the needs of the family members in their own right. Although most of these interventions are from the developed world (e.g., Community Reinforcement Approach and Family Training [CRAFT] (Archer et al., 2020), the limited evidence on interventions for or involving families in LMIC indicates the benefits of such interventions to the family and leads to better overall outcomes (Rane et al., 2017).

Case study 4 describes a trial from India in which a dyadic relapse prevention (DRP) was compared with usual care as well as individual relapse prevention in men with alcohol dependence (Nattala et al., 2010).

Case study 4: The dyadic relapse prevention (DRP) sessions were interactive, with both the family member and patient participating actively in various practice exercises. These included identifying drinking triggers in the participants and formulating a plan of action to deal with triggers, participating in role-playing, such as the rehearsal of drink refusal skills, practising problem-solving techniques, and providing family members with skills related to supporting abstinence. The dyads attended 8–10 (2–3 per week) sessions, with each session lasting for approximately an hour. The DRP was demonstrated to be superior to usual care as well as individual relapse prevention in reducing drinking behaviours and other outcomes such as family problems.

### Digital interventions

More than 80% of the population in many low-income countries in Africa, Central America, and South Asia have mobile phone subscriptions; and mobile devices account for 66–82% of web traffic in LMICs such as India, Indonesia, Nigeria, and South Africa (Naslund et al., 2017). This increasing affordability and accessibility of digital technologies in LMICs allows for an unique opportunity to harness the advances in these technologies to increase access to care.

There is emerging evidence from a range of LMICs demonstrating the role of digital technologies in diverse interventions for AUDs. This includes an online course to enhance health professionals’ knowledge about the clinical management of alcohol misuse (Pereira et al., 2015), telephone-based brief motivational intervention for reducing alcohol consumption (Wongpakaran et al., 2011; Signor et al., 2013), online self-help programme for reducing alcohol consumption among harmful or hazardous users (Andrade et al., 2016) and an online motivational intervention for preventing general substance misuse (de Oliveira Christoff and Boerngen-Lacerda, 2015). Case study 5 describes the innovative use of technology to increase access to relevant training which would allow for appropriate care for AUD to be delivered in primary care.

Case study 5: Project Extension for Community Healthcare Outcomes (ECHO) is a technology-enabled training model which has demonstrated successful outcomes globally in the management of hepatitis, chronic pain, mental health problems, and substance use disorders (Arora et al., 2010; Katzman et al., 2014; Komaromy et al., 2016; Sockalingam et al., 2018). In India, an innovative, blended training program was developed to upskill primary healthcare providers and improve compliance to AUD management (Mahadevan et al., 2020). An on-site training was complemented with videoconferencing (referred to as tele-ECHO clinics) in this program. This model facilitated easy communication between the primary care providers and the specialists using smartphones or laptops. Significant improvements were found in self-reported compliance to AUD management. This model provides the opportunity to use telementoring for providing specialised care to underserved populations and in resource-constrained settings.

Interventions leveraging digital technology will have a particularly key role in reaching individuals with AUDs in conflict zones and other hard to reach areas, and young people (nearly 90% of whom live in LMICs; UNFPA, 2014) who do not typically access clinical services but are quick to adopt new technologies. Finally, digital technologies have the potential to empower individuals with AUDs and their families to take charge of their own care and to support each other, overcoming barriers such as international borders and time zones. However, digital technology-enabled interventions for AUDs in LMICs are an emerging field, and more rigorous research is needed to evaluate the benefits of these interventions.

## Discussion

Despite the high burden of AUDs in LMICs, access to adequate and evidence-based care remains limited. There is evidence – substantial from HICs and emerging from LMICs – of a range of strategies that can be deployed to overcome access barriers and increase the penetration and coverage of interventions for AUDs even in resource-limited settings. These include designing local and culturally acceptable interventions, health systems strengthening through collaborative stepped care and task-sharing, horizontal integration of care into existing priority healthcare platforms, working with families and leveraging digital technologies. Despite the availability of such solutions, there is a long way to go and much to achieve before the treatment gap for AUDs can be reduced, both globally in general, and in LMICs in particular.

Although there is now a strong evidence base describing barriers to treatment access, the great majority of these studies have been carried out in high-income settings. More research is clearly needed to understand which of these are the most common in LMICs, as they are often influenced by contextual factors such as explanatory models and socio-economic factors, such as poverty and access to social welfare benefits. In summary, our understanding of barriers to care for AUDs in LMICs is still rather limited, and further high-quality research is needed to examine the contextual forces that drive access to treatment.

Services for people affected by AUDs need to be based on robust research evidence. Translation of research evidence into change in practice takes time (it takes 17 years for just 14% of original research to benefit practice) and the effects of interventions tested in rigorous trials are diluted when implemented at scale (Balas and Boren, 2000; Parry et al., 2013). Much of the failure of the trial effectiveness of an intervention to translate into equivalent outcomes in the real world is due to the complex and relatively uncontrollable nature of the systems within which it has to be implemented (De Savigny and Adam, 2009). Hence, research needs to move beyond testing interventions for AUDs in highly controlled conditions to addressing questions of implementation, including strategies that address the systems within which the intervention is delivered, the regulatory and funding environment, the political milieu that influences health service delivery, and societal explanatory models that affect access to care (De Silva and Ryan, 2016).

Alcohol use is complex and acts both as a mediating factor in the causal chain linking social determinants (e.g., poverty) to a range of end-point health conditions and outcomes (e.g., tuberculosis), and has its own, direct end-point health conditions as well, that is, AUDs. Additionally, alcohol use disproportionately impacts the poor and marginalised through its interaction with malnutrition and other aspects of living situations, such as overcrowding. Finally, alcohol use can lead to inequitable and differential social and economic consequences, including loss of earnings, unemployment, family disruptions, and interpersonal violence (Schmidt et al., 2010). Responsiveness to these complexities associated with AUDs require new models of care such as ‘differentiated service delivery’ (DSD), an approach used for HIV care (Ehrenkranz et al., 2019). DSD is a person-centred approach which tailors services to the specific needs of diverse groups of people. This includes more-intensive care for groups such as those naïve to treatment and those needing frequent follow-up; and less-intensive approaches for those doing well on treatment and requiring less frequent visits to health facilities. Thus, DSD places the patient at the centre of service delivery; and is a potentially suitable model for AUD care as those with AUDs include a heterogenous group with a range of conditions of varying severities, co-morbidities and diverse needs.

Historically, interventions for AUDs in LMICs have focussed on tertiary treatments and neglected promotion and prevention efforts. The latter is particularly crucial when targeting young people to delay or prevent the initiation of alcohol use. Universal prevention approaches include policy interventions primarily focused on universally reducing access to alcohol use and related injuries and harms. Examples of such policies include pricing of alcohol, as there is a consistent moderately strong association between higher taxes on alcohol and lower heavy drinking, restrictions on ‘happy hours’ which is effective in reducing heavy drinking in some populations such as college students, restrictions on the hours of operation of premises that sell alcohol which have been particularly effective in reducing alcohol-related traffic accidents, restrictions on alcohol outlet density, stringent and consistent application of drunk driving laws, and zero-tolerance laws for underage drinkers (Sher et al., 2011). In addition, promotion and prevention strategies can be delivered in settings such as schools as well as across communities. Some components of community-based approaches, albeit with modest effectiveness, include media campaigns, citizen monitoring, youth outreach programs, and server training programs (Sher et al., 2011). Finally, selective approaches are especially critical in high-risk populations such as young people and include strategies such as social norms marketing (provision of information to correct misperceptions regarding peer drinking behaviour), expectancy challenge interventions (provision of accurate information to correct misperceptions of alcohol’s effects), and harm reduction approaches to avoid excessive consumption and to minimise harmful consequences of intoxication such as drinking moderation skills and behavioural alternatives to high-risk alcohol-related behaviours (Sher et al., 2011).

Evidence of prevention strategies from LMICs is limited and primarily focused on increased taxation of alcohol, bans on alcohol advertising, restrictions on access to alcohol, and enforcement of drinking and driving legislation (Petersen et al., 2016). However, in LMICs, raising taxes is less effective if there are low levels of alcohol consumption, surrogate advertising is commonly used to market alcohol, and regulations to reduce access are ineffective when alcohol can be easily acquired through the unregulated market or brewed at home (Petersen et al., 2016).

Finally, some additional key points that need to be addressed in future research and program implementation in LMICs include testing of interventions for multiple/polysubstance use and for AUDs comorbid with mental health conditions such as depression, evaluation of policy interventions such as increased taxation on alcohol, focus on special populations (e.g., adolescents, pregnant women, indigenous groups) with AUDs, building capacity for AUD care planning and practice, increased multi-sectoral collaboration, and user-involvement and co-production approaches in designing and implementing services for AUD.

Our review has some limitations which are inherent to literature reviews. Unlike a systematic review, our literature review is not replicable as it did not follow a predefined and fixed methodology. There is a potential for selection bias as included studies may not be representative of the entire evidence base. Finally, we did not conduct a quality appraisal of included studies and treated all evidence as equally valid. Despite these limitations, a literature review such as this may sometimes be the best methodological tool especially when the aim is to provide an overview of a certain topic, to examine the state of knowledge on that particular topic, and to identify gaps in research (Snyder, 2019).

## Conclusion

Reduction of the treatment gap for AUDs in LMICs needs to be built on a foundation where there is emphasis on evidence-based decision-making, responsiveness to context and culture, and shared ownership and contributions from a range of stakeholders. This will require innovative thinking, leadership, and harnessing of synergies across multiple sectors, framing of treatment for AUDs as a public health and social development priority, and leveraging political will to support sustainable change.

## Data Availability

Data availability is not applicable to this article as no new data were created or analysed in this study.
