# Supplementary material for: Closing the treatment gap for alcohol use disorders in low- and middle-income countries
Source: Glob Ment Health (Camb). 2022 Dec 9;10:e3. doi: 10.1017/gmh.2022.57 (PMC9947611; doi:10.1017/gmh.2022.57)
Supplement: Peer review history

# Peer review: Author comment: The environmental impact of data-driven precision medicine initiatives — R1/PR1

**DOI:** 10.1017/pcm.2022.1.pr1

**Authors:** Abhijit Nadkarni

No accompanying comment.

# Peer review: Review: The environmental impact of data-driven precision medicine initiatives — R1/PR2

**DOI:** 10.1017/pcm.2022.1.pr2

**Affiliations:** University of Zimbabwe, Zimbabwe

*Comments to Author*: This is well written and an important subject. Well done.

# Peer review: Review: The environmental impact of data-driven precision medicine initiatives — R1/PR3

**DOI:** 10.1017/pcm.2022.1.pr3

**Affiliations:** New York State Psychiatric Institute, United States

*Comments to Author*: The authors addressed the feedback provided by reviewers.

# Peer review: Recommendation: The environmental impact of data-driven precision medicine initiatives — R1/PR4

**DOI:** 10.1017/pcm.2022.1.pr4

**Affiliations:** Program on Forced Migration and Health, Columbia University Mailman School of Public Health, United States

No accompanying comment.

# Peer review: Decision: The environmental impact of data-driven precision medicine initiatives — R1/PR5

**DOI:** 10.1017/pcm.2022.1.pr5

No accompanying comment.

# Peer review: Decision: The environmental impact of data-driven precision medicine initiatives — R1/PR6

**DOI:** 10.1017/pcm.2022.1.pr6

**Affiliations:** London School of Hygiene & Tropical Medicine, United Kingdom of Great Britain and Northern Ireland

No accompanying comment.
